# Pediatric stroke risk and neurotrauma from roller coasters in amusement parks

**DOI:** 10.1111/ped.70221

**Published:** 2025-10-04

**Authors:** Tomoki Morikawa, Takafumi Obara, Tsuyoshi Nojima, Kohei Tokioka, Atsunori Nakao, Kohei Tsukahara

**Affiliations:** ^1^ Department of Emergency Critical Care, and Disaster Medicine, Faculty of Medicine, Dentistry, and Pharmaceutical Sciences, Okayama University Okayama Japan

**Keywords:** amusement parks, brain injuries, carotid artery dissection, stroke, vertebral artery dissection

## Abstract

Although rare, neurotrauma has been documented as a potential risk of high‐speed, high‐acceleration amusement park rides such as roller coasters. These attractions generate rapid acceleration, deceleration, sharp turns, and significant gravitational forces, which may stress the central nervous system and cerebrovascular structures. This review analyzed pediatric stroke cases (children 15 years old or younger) linked to roller‐coaster rides reported in PubMed and summarized the key mechanisms and clinical features associated with such neurotrauma. Documented complications include internal and vertebral carotid artery dissections, with or without stroke, subdural hemorrhage, intraparenchymal hemorrhage, and post‐traumatic migraines. The aim of this review is to alert healthcare providers to the possibility of stroke induced by roller‐coaster rides, emphasizing the importance of timely diagnosis and management to prevent adverse outcomes. Key considerations include the recognition of risk factors, public education on potential risks, and strategies for preventing complications in at‐risk populations. Although intracranial hemorrhage from roller‐coaster rides is rare, individuals with predisposing conditions, such as prior head trauma or vascular abnormalities, should be evaluated carefully when presenting with neurological symptoms after such activities.

## INTRODUCTION

The development of modern amusement parks has introduced high‐speed rides, leading to a new category of complications involving indirect trauma from acceleration.^1^ Roller coasters, in particular, expose riders to sudden hyperextension, hyperflexion, and rotational forces of the neck, which can result in central nervous system injuries and vascular trauma, including damage to carotid or vertebral arteries. Although neurologic complications following roller‐coaster rides are not common, they can be severe and life threatening when they do occur.^2^, ^3^ Reported cases include cervicocephalic arterial dissection, subdural hematoma, intraparenchymal hemorrhage, subarachnoid hemorrhage, and carotid artery thrombosis with stroke.^4^, ^5^, ^6^, ^7^, ^8^, ^9^, ^10^ These cases may result from the intense forces generated during high‐speed rides, leading to arterial dissections or altered blood flow.

This review aims to increase awareness among clinicians, especially pediatricians, general practitioners, and emergency physicians involved in pediatric care, regarding injuries associated with amusement park rides and other experiences involving forceful, excessive, or repetitive head and neck movements.

## REPORTED CASES AND STUDIES

We comprehensively searched the PubMed database to find studies published from 1994 to 2024 on pediatric trauma caused by roller‐coaster rides. We conducted the search on November 1, 2024, in accordance with the Preferred Reporting Items for Systematic Reviews and Meta‐Analyses (PRISMA) guidelines. Since this study was a literature review of previously published data, it was determined that ethical approval was not required. The search terms used included “roller coaster,” and “trauma,” or “injury.” Boolean operators (AND, OR) were applied to combine search terms, and Medical Subject Headings (MeSH) were incorporated when appropriate. After our review of the previous literature, we found 24 documented cases of cerebrovascular accidents in amusement parks with available detailed information, seven of which involved children (15 years old or younger)^4^, ^5^, ^6^, ^7^, ^8^, ^9^, ^10^ (Table 1).

**TABLE 1:** Pediatric infarctions, arterial dissections, and trauma associated with roller‐coaster rides.

Year	Authors	Journal	Age, sex	Days after ride	Past medical history	Symptoms	Diagnosis	Treatment	Outcome	References
2019	Tseng et al.	Pediatr Emerg Care	12, female	2 days	None	New‐onset seizure and hemiplegia	Subdural hematoma	Conservative	Well	4
2017	Whitcomb et al.	Clin Pediatr	13, female	1 day	None	Altered mental status	Cerebral infarction caused by left internal carotid artery dissection	Craniectomy and external ventricular drainage	Facial palsy with hemiplegia	5
2017	Baumgartle et al.	Children (Basel)	12, male	1 day	None	Headache, right facial drooping, slurred speech, and dragging his right leg	A left middle cerebral artery stroke	Anticoagulants	Well	6
2015	Sorrentino et al.	Pediatr Emerg Care	8, male	2 days	None	Headache, facial droop, slurred speech, right‐sided weakness	Dissected right vertebral artery embolus	Anticoagulants	Well	7
2015	Nouh et al.	Pediatr Neurol	4, male	2 days	None	A left facial droop	Internal carotid artery dissection	Aspirin	Well	8
2006	Roldan‐Valadez et al.	Eur J Pediatr Neurol	13, female	2 weeks	None	Headache, right‐sided paresis	Subdural hematoma	Surgery	Well	9
2001	Lascelles et al.	J Neurol Neurosurg Psychiatry	11, male	Immediately	None	Headache	Left vertebral artery dissection	Anticoagulants	Well	10

The patients' clinical characteristics included a predominance of teenagers, who comprised over 70% (5/7) of the cases and were able to ride roller coasters without age or height restrictions. All individuals were previously healthy with no significant past medical history. Most patients developed symptoms relatively early, within two days of riding, headache being the most common complaint, reported in four cases. Acute subdural hematoma occurred without evident overt head trauma, and one case required surgical intervention. Additionally, in a case of stroke due to dissection, one patient developed facial palsy with hemiplegia; excluding this case, outcomes were favorable without complications. There were cases where individuals rode multiple times, but there were also cases where they rode only once.

## DISCUSSION

Roller coasters generate substantial gravitational forces (G‐forces) during rapid acceleration, deceleration, and directional changes. These forces can place stress on the central nervous system, potentially contributing to traumatic injuries. High G‐forces may result in cerebral injury, including subdural hematoma or intracerebral hemorrhage, through vascular shearing mechanisms. Furthermore, abrupt speed and directional changes could induce a whiplash effect, leading to cervical spine injuries or related strains.^11^, ^12^ Upon investigating cases in Japan, roller coasters were found to have the following specifications: height differences of 79 m, maximum speeds of 172 km/h (reached in 1.8 s), ride durations of three minutes and 36 s, maximum inclines of 90°, and maximum G‐forces of 4.3G. These specifications rank one ride 10th globally in height difference and 4th in maximum speed.^11^, ^13^

In individuals with prior head trauma, rotational acceleration forces during roller‐coaster rides could disrupt existing adhesions between the cerebral cortex and dura, potentially resulting in vascular rupture. Additionally, repeated exposure to acceleration and deceleration forces may overstretch the bridging veins, predisposing riders to subdural bleeding. In extreme cases, these repetitive forces might cause intimal tears to the cervical internal carotid artery, leading to thromboembolic events. The precise threshold for tolerance to repetitive rotational stresses and G‐forces in otherwise healthy individuals, as well as in those with preexisting conditions, remains uncertain.^8^

Regarding the possibility that repeated roller‐coaster rides causes the rupture of bridging veins, it is known that in shaken baby syndrome, shaking motions, often due to abuse, lead to the rupture of bridging veins.^14^ In this mechanism, the dura mater remains attached to the skull, while the arachnoid and bridging veins are relatively firmly attached. In contrast, the attachment between the dural border cell layer and the bridging veins is weaker. As a result, stress is concentrated on the dural border cell layer, causing it to tear and separate. Blood accumulates in the opened dural border cell layer, furthering its separation and leading to the progressive formation of a hematoma.

Prior investigations have shown that G‐forces alone may not be sufficient to cause intracranial injury.^15^ Experimental studies using accelerometers to measure linear and rotational forces on the head during roller‐coaster rides have demonstrated that the loads experienced are comparable to those in activities such as soccer headers and significantly lower than those encountered in boxing or American football. However, such data are limited to healthy individuals and may not be applicable to those with underlying medical conditions or a history of brain injury.

Interestingly, transient high linear accelerations, such as those generated during sneezing (up to 10G), are unlikely to cause harm due to their brief duration (approximately 0.002 s). In contrast, sustained exposure to 5–9G for over 40 s, as seen in fighter pilots, has been associated with loss of consciousness.^15^ Understanding the risk of traumatic brain injury requires a comprehensive assessment of head motion parameters, including (1) the direction of movement, (2) the magnitude of velocity and acceleration, and (3) the duration of force application. These factors are crucial for evaluating the potential for trauma, particularly in cases involving repetitive high‐intensity activities such as roller‐coaster rides.

Neurotrauma in children encompasses a range of conditions, including concussion, acute subdural hematoma, cervical spine injury, and vertebral artery dissection. A concussion is a form of mild traumatic brain injury that can occur from sudden impact or rapid movement, presenting symptoms such as dizziness, headache, or confusion. Acute subdural hematoma can be categorized into two types: one type involves bleeding into the subdural space due to the rupture of cortical vessels associated with cerebral contusion, while the other type involves bleeding caused by the rupture of bridging veins without primary brain parenchymal injury. Cervical spine injury is of particular concern in children, who are more vulnerable due to the anatomical immaturity of the pediatric cervical spine. Full development of the cervical spine is not achieved until approximately 8–10 years of age.^7^ The combination of ligamentous laxity, shallow and angled facet joints, and underdeveloped vertebral bodies in children allows for greater neck hypermobility. This instability is further exacerbated by the relatively larger head size and weaker neck musculature in pediatric patients. Consequently, high‐speed rides with sudden movements can cause cervical strain, leading to neck pain or, in rare cases, more severe spinal injury.

Additionally, although strokes are relatively uncommon in children, vertebral artery dissection is a recognized cause of pediatric stroke.^16^ Cervicocephalic arterial dissections account for approximately 2% of ischemic strokes in the general population and 5% of ischemic strokes in pediatric cases.^17^ Rapid head movements can occasionally lead to vertebral artery dissection, resulting in symptoms such as headache, dizziness, or even stroke‐like events. Sudden changes of direction and acceleration might cause indirect trauma to mobile portions of the cervicocephalic arteries and lead to intimal tears.

Early diagnosis is critical to attain better outcomes, especially when the presentation is asymptomatic or vague. Particularly, dissection should always be included in the differential when patients, whether young or old, present ischemic symptoms without typical stroke risk factors. Patients recovering from strokes after dissection are usually started on anticoagulants to prevent recurrence. Early diagnosis of dissection, especially before the onset of infarction, is associated with a more favorable prognosis.^10^

Generally, patients in emergency centers and outpatient clinics often report symptoms like tension headaches, migraine, and musculoskeletal or myofascial pain, which are typically attributed to benign conditions. Consequently, the diagnosis of traumatic injury is frequently misdiagnosed or missed. Serial changes in angiographic appearance, as observed in adult cases, lead to the correct diagnosis of artery dissection.^18^ Axial T1‐weighted cervical magnetic resonance imaging combined with magnetic resonance angiography is preferred because it is a noninvasive, highly sensitive test. It is possible that individuals with preexisting cervical spine problems, vascular disorders, or intracranial abnormalities may be at higher risk for neurotrauma. Considering that patients experience headaches and become symptomatic after riding roller coasters, one possible explanation is the presence of adhesions between the cortical surface and the dura mater due to a previous head injury.

Amusement parks typically post warnings for certain categories of riders, such as those who are elderly or very young, those with cardiac conditions, and pregnant women. Although roller coasters adhere to established safety standards, this review highlights that rapid acceleration, deceleration, and abrupt ascents and descents can significantly impact the head, particularly in individuals with preexisting conditions. While the risk of stroke from roller‐coaster rides is very low in healthy individuals, awareness of complication rates remains important.

The competitive amusement park industry continues to push the boundaries of speed and height in roller‐coaster design. Modern roller coasters incorporate advanced safety measures to mitigate risks associated with high G‐forces and rapid movements. Ongoing research seeks to better understand the physiological effects of extreme accelerations to further enhance safety standards. Park operators routinely monitor G‐force limits to ensure rides remain within safe parameters for the general population, based on experimental data and evaluations. Although pediatric amusement park ride‐associated neurotrauma cases are rare, it is important that individuals experiencing warning symptoms after a ride seek prompt medical attention to prevent severe neurological complications. It is also recommended that those with known risk factors for heart disease consult a healthcare provider before participating in high‐intensity activities such as roller‐coaster rides.^19^ Persistent headaches and/or neck pain after roller coaster rides should alert the practitioner to these rare complications.^20^, ^21^

## CONCLUSION

While serious neurotrauma from roller‐coaster rides is rare, certain individuals may be at greater risk due to underlying health conditions or vulnerabilities. Awareness, precautionary measures, and adherence to ride safety guidelines are essential for minimizing risks.

## AUTHOR CONTRIBUTIONS

T.M. and T.O. wrote the manuscript. T.N, K.T, A.N, and K.T. supervised and critically revised the manuscript. All authors met the criteria for authorship contribution based on the recommendations from the International Committee of Medical Journal Editors (ICMJE). All authors read and approved the final manuscript.

## CONFLICT OF INTEREST STATEMENT

The authors declare no conflict of interest.

## ETHICS STATEMENT

As this study was a literature review, it was determined that ethical approval was not required.
